# Deficits in motor and cognitive functions in an adult mouse model of hypoxia-ischemia induced stroke

**DOI:** 10.1038/s41598-020-77678-8

**Published:** 2020-11-26

**Authors:** Li Feng, Chun-Xia Han, Shu-Yu Cao, He-Ming Zhang, Gang-Yi Wu

**Affiliations:** 1 School of Life Sciences, South China Normal University, Guangzhou, 510631 China; 2 Institute for Brain Research and Rehabilitation, South China Normal University, Guangzhou, 510631 China

**Keywords:** Neuroscience, Cell death in the nervous system

## Abstract

Ischemic strokes cause devastating brain damage and functional deficits with few treatments available. Previous studies have shown that the ischemia-hypoxia rapidly induces clinically similar thrombosis and neuronal loss, but any resulting behavioral changes are largely unknown. The goal of this study was to evaluate motor and cognitive deficits in adult HI mice. Following a previously established procedure, HI mouse models were induced by first ligating the right common carotid artery and followed by hypoxia. Histological data showed significant long-term neuronal losses and reactive glial cells in the ipsilateral striatum and hippocampus of the HI mice. Whereas the open field test and the rotarod test could not reliably distinguish between the sham and HI mice, in the tapered beam and wire-hanging tests, the HI mice showed short-term and long-term deficits, as evidenced by the increased number of foot faults and decreased hanging time respectively. In cognitive tests, the HI mice swam longer distances and needed more time to find the platform in the Morris water maze test and showed shorter freezing time in fear contextual tests after fear training. In conclusion, this study demonstrates that adult HI mice have motor and cognitive deficits and could be useful models for preclinical stroke research.

## Introduction

Stroke is a leading cause of death and long-term disability in adults worldwide^1^. Ischemic stroke is mainly caused by atherothrombosis of large cervical and intracranial arteries or by embolism from the heart. It is the most common type of stroke, contributing to 78–85% of total patients, but few treatments are available^2–5^. Many drug candidates were effective in ischemic animal models but failed in stroke patients, calling for better practice of preclinical studies and new animal models^6^.

As the most commonly-used animal stroke model, the transient middle cerebral artery occlusion (MCAO) model only represents a small part (2.5–11.3%) of all large vessel occlusion (LVO) stroke patients^7^. But in the majority of LVO patients, occluded arteries were not recanalized and the infarct zone had limited blood perfusion^7^. Thus, a more relevant animal model and better practice in preclinical studies are urgently needed ^6,7^.

Previous reports showed that adult HI model is closer to the clinic situations^8,9^. This model usually combines permanent ligation of unilateral common carotid arteries with a period of hypoxia of 6–8% O_2_. The rodent HI model was first established in new-born rat pups^10^, and then modified and widely used in the 1980s to mimic perinatal hypoxic-ischemic brain damage^11–13^. Recently, it has been successfully applied to adult mice^8,14^. Unlike in the widely used MCAO model, ischemia-followed hypoxia rapidly induces clinically similar thrombosis in the adult HI model^8,9^. Previous reports showed that the endothelial cells became rapidly activated and induced spontaneous thrombosis of blood vessels in the injured side of striatum and cortex about 1 h after ischemia-hypoxia^9^.

It is well documented that during ischemic injury, ischemia rapidly causes the death of neurons^15,16^ and irreversibly induces the disruption of neuronal dendrites and synaptic structures in the injured core^17^. Meanwhile, the resting glial cells would transform into reactive status to produce post-ischemic inflammatory responses^18,19^. In the long run, the reactive astrocytes form the glial scar in the injured zone and the reactive microglial cells contribute significantly to the secondary neuronal injury by secreting harmful inflammatory factors^15,20^. Most recent studies of the adult HI model are limited to the short-term neuropathology and molecular mechanisms^8^, but long-term pathology and behavioral deficits remain largely unknown. To answer these questions, we first used FJB staining^16,21^ to confirm the early stage of neuron degeneration, and then used immunostaining to examine the long-term neuronal loss (NeuN^+^), dendrite damage (MAP2^+^)^17^ and reactive glial cells (GFAP^+^ or IBA1^+^)^15^ in the adult HI mice. We then performed a series of behavioral tests to demonstrate that the adult HI mice have short-term and long-term motor and cognitive deficits.

## Results

### Survival rate and scores in adult hypoxic and ischemic mouse model

Sixty male C57BL6 mice (12- to 14-weeks old) were subjected to permanent ligation of the right common carotid arteries (RCCA) and then underwent 40-min hypoxia as previously reported^8^. 36 mice survived the procedure with a 60.0% survival rate consistent with a previous report^8^. 25 mice were used as sham control. Among them, 3 pairs of sham and HI mice were only used for the pilot experiments specifically designed for pathological validation. For the behavioral tests, we used 22 sham mice and 33 stroke mice to train and screen before surgery. As similar exclusion^22,23^, 3 mice in the sham and 5 mice in the stroke group showed poor performance after training, and 9 mice were too weak at 24–48 h after HI condition, all of which were excluded from further tests. Therefore, 19 pairs of sham and stroke mice from 3 different cohorts were used for further behavioral experiments. The stroke mice began showing behavioral deficits after HI on the modeling day and were scored about 30 min after hypoxia as previously reported (also Methods)^8^. In our study, 15 (79.0%) mice showed a circling performance (grade 3), 2 (10.53%) lay motionless (grade 4) and 2 (10.53%) had ptosis of the eyelid (grade 2) after stroke.

**Figure 1:**
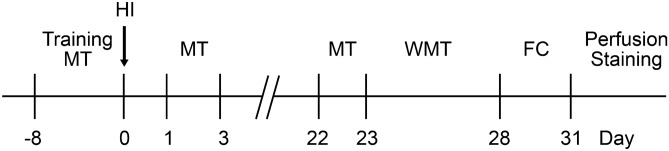
Schematic illustration of the experiments. Abbreviations: MT, motor test; HI, stroke modelling of ischemia followed hypoxia (HI); *WMT* water maze test, *FC* fear conditioning test.

As shown in Fig. 1, for the behavioral tests, mice were pre-trained for several successive days and examined for baseline motor performances. After surgery, the motor and cognitive deficits were measured after 24–48 h as the short-term and 3–4 weeks as the long-term changes in behaviors.

### Neuron losses were found in the ischemic striatum and hippocampus

**Figure 2:**
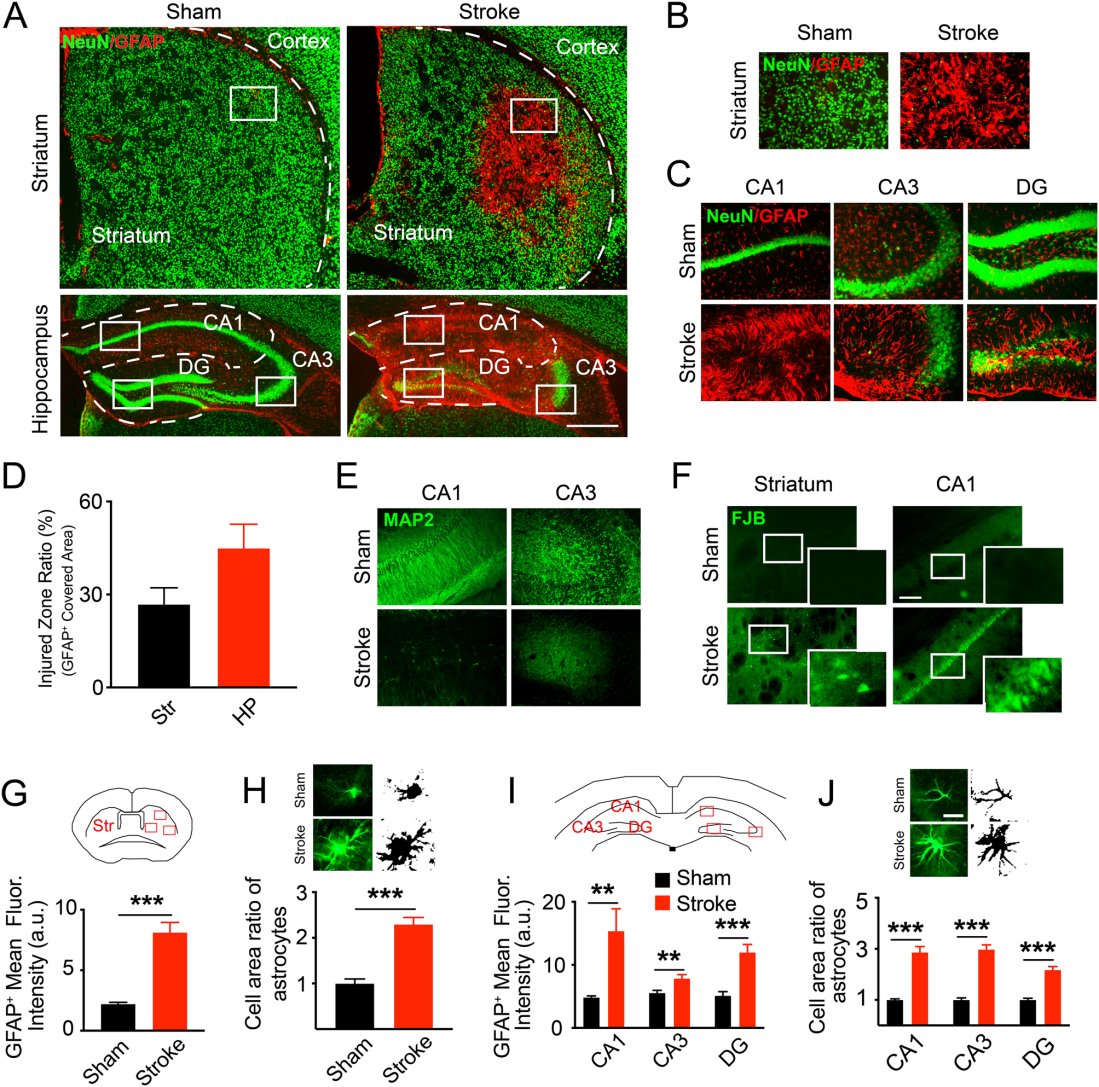
Pathology in the striatum and hippocampus of adult ischemia and hypoxia (HI) mice. (**A**) Overview of the pathology in striatum and hippocampus of sham and HI mice at 4 weeks after HI conditions. Obvious neuron loss (stained with a neuronal marker, NeuN, green) and reactive astrocytes (stained with an astrocytic marker, GFAP, red) are in these two areas. (**B**,**C**) The magnified images of the injured area in striatum (**B**) and hippocampus (**C**). (**D**) Quantification data of the injured area, calculated as the GFAP^+^ overexpressed area ratio with the total area of the striatum and hippocampus. N = 5 mice with 4 slices per animal. Abbreviations: Str, striatum; HP, hippocampus. (**E**) showing the scarce expression of another neuronal marker, MAP2 (green), on the neuronal dendrites in the ischemic CA1 and CA3 areas, indicating the disruption of synaptic connections. (**F**) Represent the neuron degeneration by FJB^+^ cells (green) in ischemic striatum and CA1 area 24 h after HI conditions. (**G**)–(**J**) Data showed the morphological changes of astrocytes in HI mice 4 weeks after stroke. (**G**,**I**) Quantification of the significant increase in GFAP^+^ expression in HI group than sham. Unpaired t-test, **P < 0.01, ***P < 0.001. N = 3 mice in each group with 3 slices per animal. Data are collected from three fields in striatum and one field in each hippocampal sub-region shown in cartoons. (**H**,**J**) Fluorescent sample images and cropped cells with binary images showed the hypertrophy status of reactive astrocytes by larger cell area in the ischemic striatum (**H**) and CA1 (**J**). Unpaired t-test, **P < 0.01, ***P < 0.001. For area analysis, N = 15 cells in each slice with 3 slices per animal. The images were all captured by the fluorescent microscope (Nikon Eclipse Ni). The cropped cells with binary images in (**H**,**J**) were performed in Image J 2.0.0 (FIJI). Quantitative analysis of the fluorescence intensity and morphological changes were performed in FIJI. Data are shown as the mean and SEM. Scale bars in (**A**) represent 500 μm, (**F**) 100 μm and (**J**) 10 μm.

We first examined the morphology of brain injuries in adult HI mice. To confirm the feasibility of modeling conditions, four representative brain slices from one pair of mice were used to detect neuronal degeneration at 24 h post stroke via FJB staining^8,21^. We found obvious FJB^+^ cells scattered in the ipsilateral striatum, CA1 and CA3 pyramidal layers, and DG hilus area (Fig. 2F), showing neuronal degeneration at an early stage after ischemia and hypoxia, which is similar to the previous report^8^.

We then focused on long-term neuron losses and reactive gliosis^24^ in adult HI models using immunofluorescent staining for selective markers. Four representative slices for each brain region of interest, spanning from + 0.4 to − 2.5 mm to the bregma, were selected from consecutive brain sections and used to analyze the morphological changes at 4 weeks after a stroke. The immunoreactivity of the neuron specific marker NeuN^+^ was commonly lost in part of the ipsilateral cerebral cortex (Supplementary Fig. 1A). The central area of the striatum and parts of the pyramidal layers of CA and DG granular layers showed an almost complete loss of NeuN (Neuronal Nuclei, a neuron specific marker) immunoreactivity (Fig. 2A–C) concomitant with a dramatic increase in reactive astrocyte marker, GFAP immunoreactivity. We estimated the injured area by calculating the percentage of the gliosis area^25–27^ in the striatum and hippocampus of stroke mice as shown in Fig. 2D. We also quantified the relative changes in the expression of GFAP in 3 pairs of mice and found a significant increase in the striatum, CA1, CA3 and DG region of the hippocampus in HI mice compared with those of the sham (Fig. 2G,I; striatum: 2.20 ± 0.15 in sham, 8.11 ± 0.84 in stroke, ***P < 0.001; CA1: 4.83 ± 0.24 in sham, 15.35 ± 3.54 in stroke, **P = 0.0091; CA3: 5.54 ± 0.42 in sham, 7.83 ± 0.63 in stroke, **P = 0.0081; DG: 5.12 ± 0.65 in sham, 11.97 ± 1.27 in stroke, ***P = 0.0002; unpaired t-test, N = 3). Furthermore, when closely examining the morphology of the reactive astrocytes, we found that in the ischemic striatum and hippocampus, many reactive astrocytes had enlarged soma and processes (Fig. 2H,J; area ratio in striatum: 1.00 ± 0.010 in sham, 2.30 ± 0.15 in stroke, ***P < 0.001; CA1: 1.00 ± 0.04 in sham, 2.86 ± 0.23 in stroke, ***P < 0.001; CA3: 1.00 ± 0.08 in sham, 2.97 ± 0.19 in stroke, ***P < 0.001; DG: 1.00 ± 0.07 in sham, 2.17 ± 0.13 in stroke, ***P < 0.001; unpaired t-test, N = 15 cells in each slice of 3 mice), and formed glia scar-like structures in the injury area (Fig. 2A–C). In addition to the loss of NeuN immunoreactivity, we also found significant losses of MAP2 immunoreactivity in the pyramidal layers of CA1 and part of CA3, suggesting degeneration of neuronal dendrites induced by ischemia (Fig. 2E).

**Figure 3:**
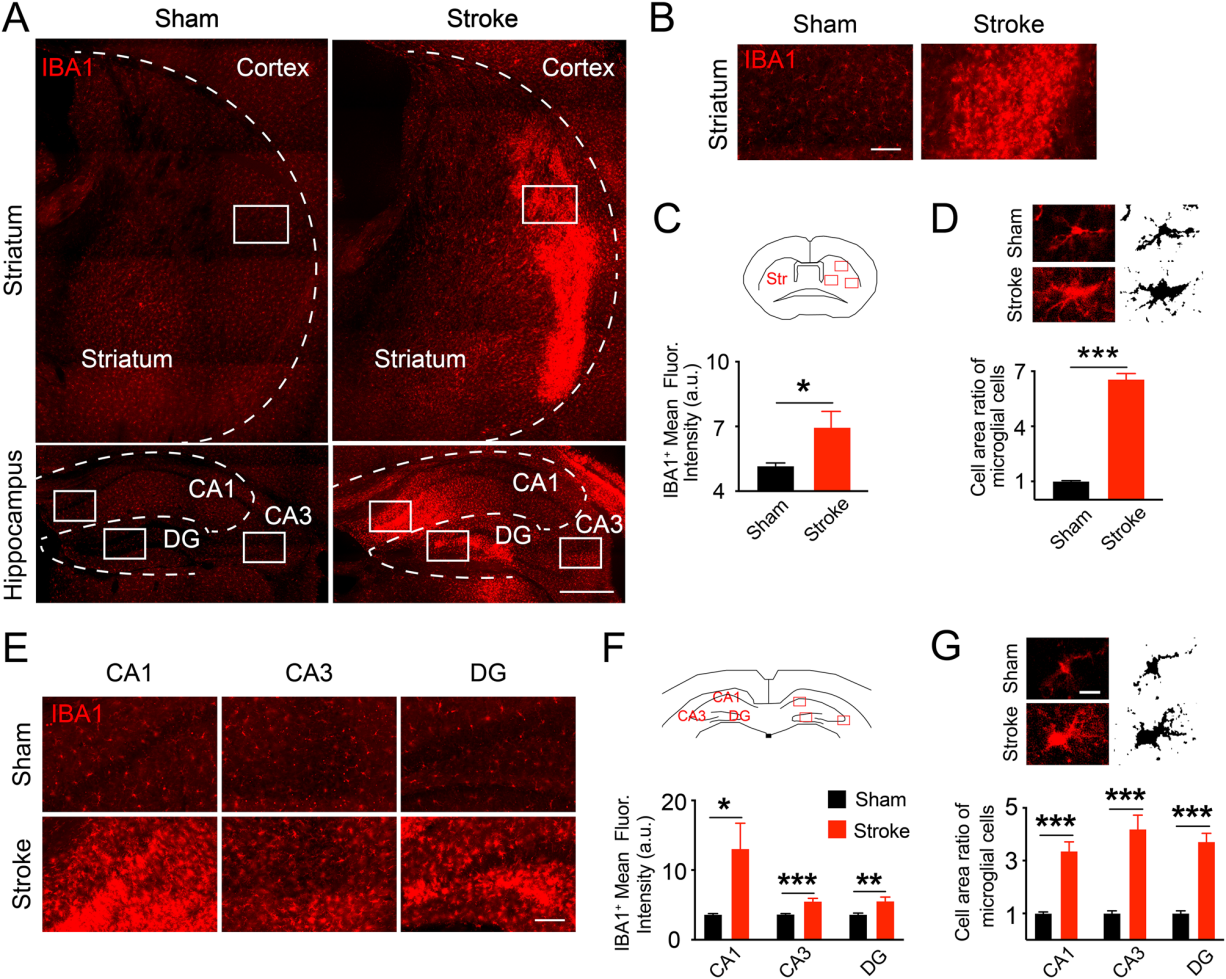
Reactive microglia in the striatum and hippocampus 4 weeks after hypoxia and ischemia. (**A**) Overview of the reactive microglia (stained by the microglial marker, IBA1^+^, red) in ischemic striatum and hippocampus. These reactive microglia could contribute to the secondary neuronal injury in the long term after HI conditions. (**B**,**E**) Representative views of the reactive microglia in the striatum and hippocampus. (**C**,**F**) The increased IBA1^+^ expressions in HI mice than sham in both areas. Unpaired t-test in (**C**,**E**), *P < 0.05, **P < 0.01, ***P < 0.001. N = 3 mice in each group with 3 slices per animal. Data are collected from three fields in striatum and one field in each hippocampal sub-region shown in cartoons. (**D**,**G**) Sample images and cropped cells with binary images showed the hypertrophy status of reactive microglia by larger cell area in the ischemic striatum (**D**) and CA1 (**G**). Unpaired t-test, **P < 0.01, ***P < 0.001. For area analysis, N = 15 cells in each slice with 3 slices per animal. The slices were observed by an inverted fluorescent microscope (EVOS FL Auto) and the cropped cells with binary images in (**D**,**G**) were performed in Image J 2.0.0 (FIJI). FIJI was also used to analyze the fluorescence intensities and morphological changes. Data are shown as the mean and SEM. Scale bar in (**A**) is 500 μm, (**E**) 100 μm and (**G**) 10 μm.

Microglia are the resident immune cells of the brain and are thought to have both beneficial and detrimental effects during ischemic stroke^15,28^. We therefore examined the microglia using immunofluorescence staining for the microglia marker IBA1 at 4 weeks after HI condition (Fig. 3A,B,E and Supplementary Fig. 1B). As shown in Fig. 3, similar to the reactive astrocytes, the expression of IBA1 in microglia also significantly increased in the ipsilateral central region of striatum and most parts of the hippocampus of the HI mice (Fig. 3C,F; striatum: 5.14 ± 0.15 in sham, 6.95 ± 0.75 in stroke, *P = 0.0325; CA1: 3.62 ± 0.14 in sham, 13.05 ± 3.68 in stroke, *P = 0.0209; CA3: 3.66 ± 0.09 in sham, 5.51 ± 0.43 in stroke, ***P = 0.0003; DG: 3.63 ± 0.19 in sham, 5.54 ± 0.58 in stroke, **P = 0.0065; unpaired t-test, N = 3). The microglia became hypertrophy with significantly enlarged cell area in HI mice compared with those in sham (Fig. 3D,G; area ratio in striatum: 1.11 ± 0.12 in sham, 6.55 ± 0.32 in stroke, ***P < 0.001; CA1: 1.00 ± 0.05 in sham, 3.36 ± 0.35 in stroke, ***P < 0.001; CA3: 1.00 ± 0.010 in sham, 4.19 ± 0.53 in stroke, ***P < 0.001; DG: 1.00 ± 0.10 in sham, 3.71 ± 0.32 in stroke, ***P < 0.001; unpaired t-test, N = 15 cells in each slice). These results suggest that the microglia became chronic reactive and might have contributed to the brain injury^28^. Taken together, our results showed significant long-term neuronal losses and reactive glial cells in the ipsilateral central region of striatum and most part of the hippocampus of the HI mice.

### Motor deficits in adult hypoxic-ischemic mice

**Figure 4:**
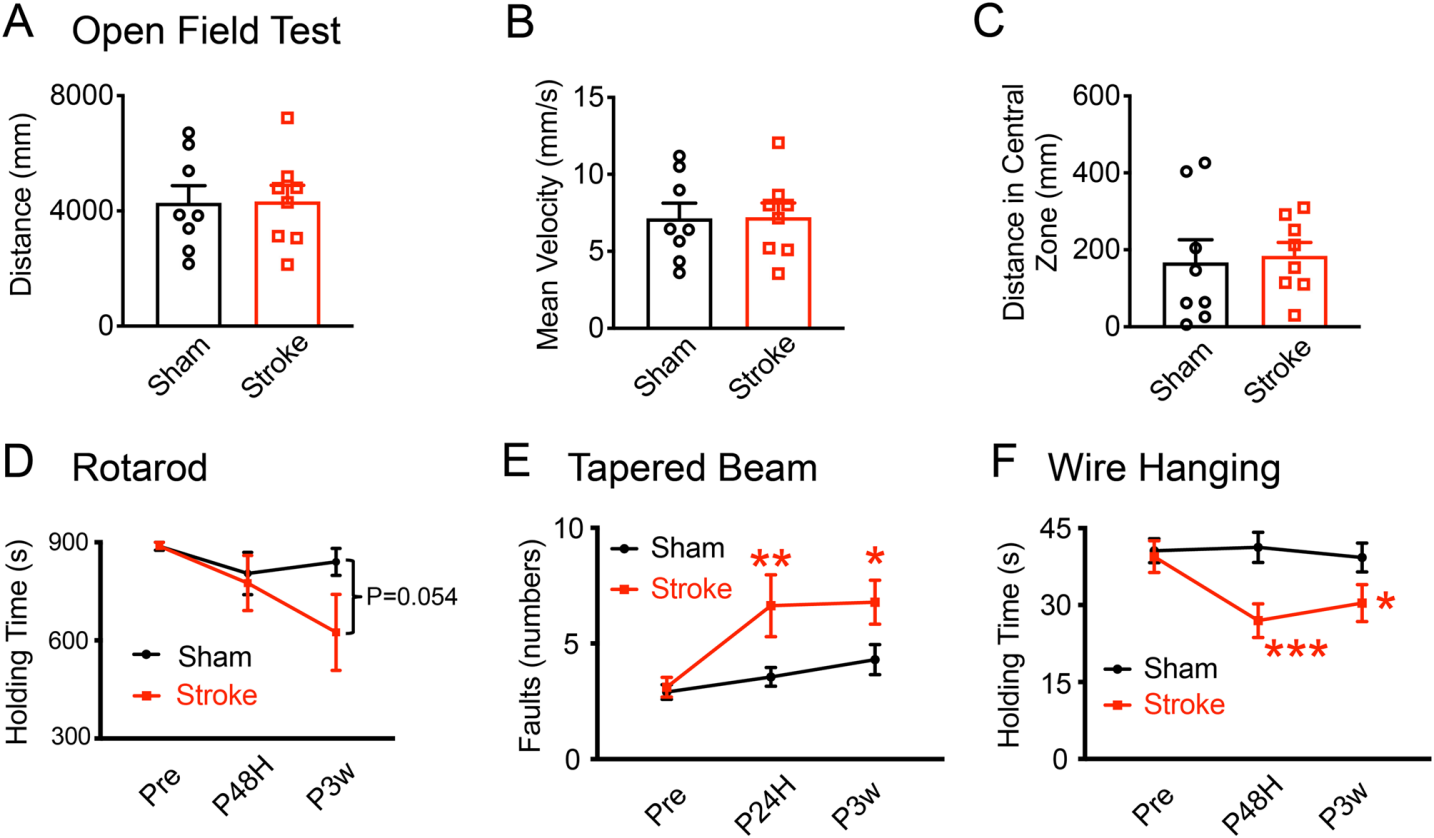
Motor behavioral deficits in stroke mice. (**A**)–(**C**) Represent the similar performances of sham and HI mice in the open field test (OFT) at 3 weeks after stroke. Parameters of the OFT include the total distance (**A**), the mean velocity (**B**) and the time spent in the center zone (**C**). N = 8 mice per group in (**A**)–(**C**). (**D**) Quantitative measurements showed comparable performances of sham and HI mice in the rotarod test. N = 14 mice in sham and N = 10 in stroke group. (**E**,**F**) Show the motor deficits of HI mice in the taper beam and wire hanging tests. (**E**) Increased fault numbers of stroke mice in tapered beam test at 24 h and 3 weeks after stroke than sham group. N = 19 mice per group. (**F**) Decrease in holding time of HI mice in the wire hanging test at 48 h post ischemia and remaining to 3 weeks after stroke. N = 19 mice per group. Data are shown as the mean and SEM for each group. * Compared with sham group, *P* < 0.05, **P < 0.01, ***P < 0.001, unpaired t-test in (**A**)–(**C**); two-way repeated ANOVA followed by fisher multiple comparison test in (**D**)–(**F**).

We then performed 4 commonly used motor behavioral tests to examine the motor deficits of the stroke mice. We pre-trained all the mice and screened out the individuals that performed poorly before surgeries or were too weak after the HI condition. To evaluate the general exploratory behaviors, the open field test (OFT) was used to test the mice at 3 weeks after stroke^29,30^. We found that the total distance (Fig. 4A; unpaired t-test, *P*_(sham vs stroke, 3w)_ = 0.9583) and the mean velocity (Fig. 4B; unpaired t-test, *P*_(sham vs stroke, 3w)_ = 0.9575) of the sham and stroke mice were comparable at 3 weeks after stroke. The time spent in the center zone (Fig. 4C; unpaired t-test, *P*_(sham vs stroke, 3w)_ = 0.6705) by the sham and the stroke mice was also similar, indicating no abnormal hyperactive exploratory behaviors in the adult HI mice. Therefore, there were no apparent differences in the general exploratory behaviors of the sham and HI mice.

The rotarod test is commonly used to measure the motor coordination and balance of the mice^30,31^. At 48 h post stroke, the sham mice were able to consistently walk on a rotating rod and rarely fell after successive training. Quantitative measurements showed that the holding time of the sham control and HI groups was comparable, although HI mice showed a slight but not significant decrease in holding time at 3 weeks after stroke (Fig. 4D; two-way repeated ANOVA, for the groups: F (1, 9) = 0.9669, *P* = 0.3511; for the group x time interaction: F (2, 18) = 1.52905, *P* = 0.2436; followed by fisher post hoc, sham vs stroke, *P*_pre_ = 0.95736, *P*_PHI48H_ = 0.56796, *P*_PHI3w_ = 0.05437). Therefore, our data show that the OFT and the rotarod are not sensitive tests for detecting motor deficits in the adult HI model.

The tapered beam test is commonly used to evaluate motor dysfunction in a mouse’s hind legs^31^. Before surgery, the mice mainly used the central board and passed the beam without many errors of stepping on the ledges (fault). However, the HI mice showed a consistent increase in the number of foot faults both at 24 h and 3 weeks after stroke (Fig. 4E). Through a two-way repeated ANOVA test, a significant effect of both group and time factors on the faults with no interaction was found (for groups: F (1, 18) = 10.4892, *P* = 0.00456; for the time: F (2, 36) = 6.6065, *P* = 0.0036; for the group x time interaction: F (2, 36) = 2.7451, *P* = 0.0777; followed by fisher post hoc, sham vs stroke, *P*_pre_ = 0.8336, *P*_PHI48H_ = 0.0038, *P*_PHI3w_ = 0.01715). Similarly, in the wire hanging test, the mice were trained to suspend their bodies from a wire with only their forelimbs^30^. Two-way repeated ANOVA analysis showed that the HI condition and the time factor have significant effects on the holding time of mice (Fig. 4F; for groups: F (1, 18) = 8.4623, *P* = 0.00936; for the time: F (2, 36) = 3.5953, *P* = 0.0377; for the group × time interaction: F (2, 36) = 3.86076, *P* = 0.03026). The holding time of the stroke mice was significantly shorter than that of sham mice at 48 h and remained decreased at 21 days after stroke, suggesting the reduced grip strength of the HI mice (by fisher post hoc test, *P*_pre_ = 0.7561, *P*_PHI48H_ = 0.0005, *P*_PHI3w_ = 0.02202). Together, these results demonstrated that the tapered beam and wire hanging tests could reliably distinguish the short- and long-term motor deficits of adult HI mice.

### Deficits of cognitive function in adult hypoxic-ischemic mice

**Figure 5:**
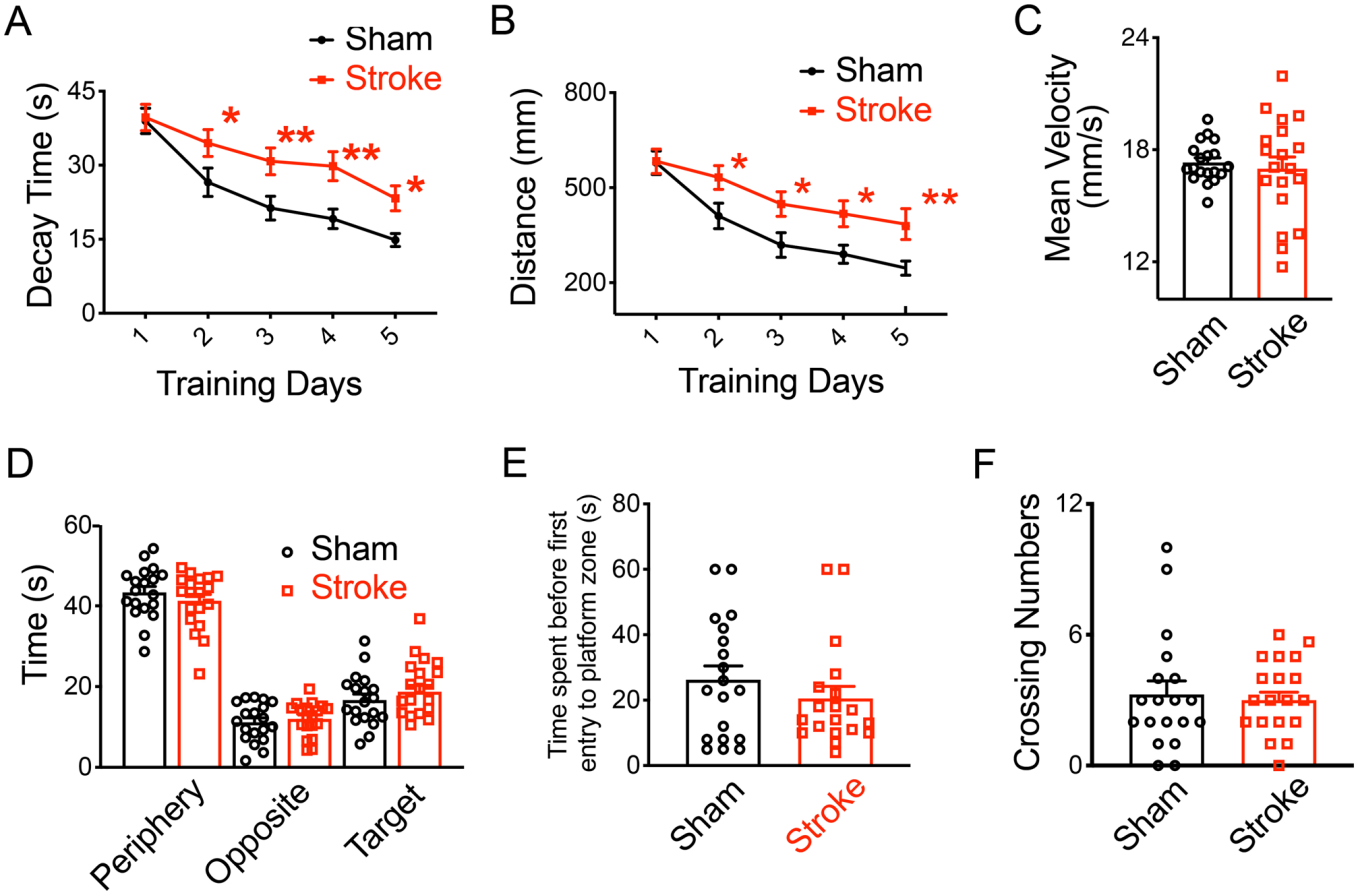
Behavioral deficits of stroke mice in the WMT. (**A**,**B**) show the cognitive deficits of HI mice in the spatial navigating training of the WMT at 4 weeks after stroke. Increased decay time (**A**) and longer swimming distances (**B**) to the platform showed in HI mice than those of sham mice in the training of the WMT. (**C**) Similar swimming speeds in sham and HI groups. (**D**)–(**F**) Sham and HI mice showed no significant differences in the probe test, including the time spent in the periphery, the opposite and the target quadrants (**D**), the time spent before the first entry to the platform zone (**E**), the crossing numbers (**F**). Data are shown as the mean and SEM for each group. Two-way repeated ANOVA followed by fisher multiple comparison test in (**A**,B) or unpaired t-test in (**C**)–(**F**), *P < 0.05, **P < 0.01. N = 19 mice per group.

Next, we further examined the long-term cognitive function of the adult HI model. The water maze test (WMT) was used to assess the hippocampus function of adult mice^29,32,33^. In the WMT, mice were trained to use the visual cues on pool walls to find a hidden platform to escape from the opaque water. We randomly placed the mice in one of the 4 start positions to make them use the visual cues in order to navigate a direct path to the platform. There was a significant effect on both of the groups and successive training factors on the decaying time with no interaction by two-way repeated ANOVA analysis^34–36^ (Fig. 5A; for groups: F (1, 18) = 16.16987, *P* = 0.0008; for the training: F (4, 72) = 25.10918, *P* = 4.87 × 10^–13^; for the group × time interaction: F (4, 72) = 1.4611, *P* = 0.223). The sham mice quickly swam to the landing platform after 5-day successive training, but the stroke mice required more time to find the landing platform at 4 weeks’ post stroke (by fisher post hoc, *P*_Day1_ = 0.83287, *P*_Day2_ = 0.01729, *P*_Day3_ = 0.00484, *P*_Day4_ = 0.00168, *P*_Day5_ = 0.01163). For the swimming distances in the WMT, we found these two factors also had obvious effects with no interaction on the distances of mice by two-way repeated ANOVA test (Fig. 5B; for groups: F (1, 18) = 10.45917, *P* = 0.0046; for trainings: F (4, 72) = 18.2075, *P* = 2.26 × 10^–10^; for the group × time interaction: F (4, 72) = 1.67237, *P* = 0.16578). Post hoc comparisons for each day of testing indicated that the swimming distances of the HI mice were significant longer than those of the sham mice (by fisher post hoc, *P*_Day1_ = 0.92827, *P*_Day2_ = 0.0182, *P*_Day3_ = 0.012, *P*_Day4_ = 0.013, *P*_Day5_ = 0.00722). The mean swimming velocities of both groups were similar (unpaired t-test, *P* = 0.6217; Fig. 5C), indicating that it was not swimming speed that delayed the stroke mice from finding the landing platform. In the probe test, we analyzed the time spent in the periphery (unpaired t-test; *P* = 0.3375; Fig. 5D), the opposite (*P* = 0.5605) and the target quadrants (*P* = 0.3375), the time spent before the first entry to the platform zone (unpaired t-test, *P* = 0.3118; Fig. 5E) and the crossing numbers over the hidden platform (unpaired t-test, *P* = 0.7200; Fig. 5F). All of these parameters were similar in sham and stroke mice, likely due to the compensatory function of the contralateral side and/or overtraining in the tests^37,38^, which needs further investigation in the future.

**Figure 6:**
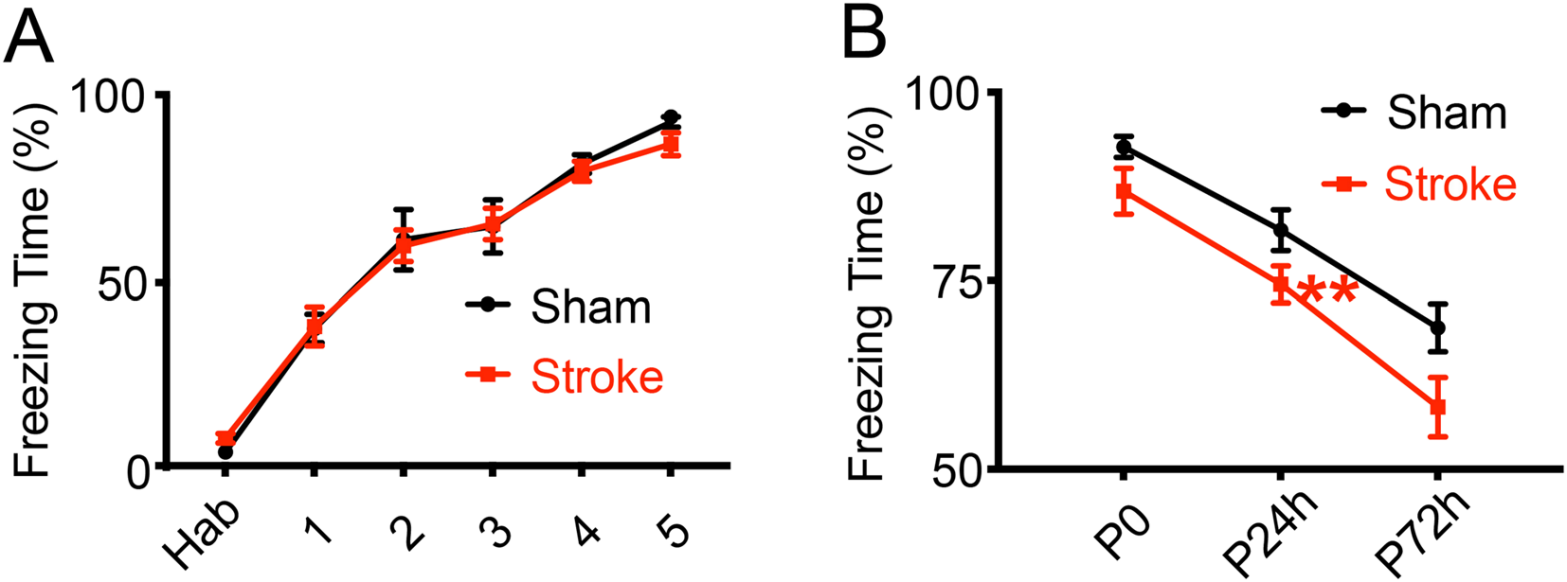
Behavioral deficits of stroke mice in the fear conditioning test. (**A**,**B**) Both the sham control and HI mice can acquire the fear association between the electronic foot shocks and the contextual cues in the fear training at 4 weeks after stroke, but not solid at 24 h after training. (**A**) The acquired fear behaviors in sham and HI mice after training by displaying high percentages of freezing responses. Habitation (hab) represents the baseline freezing percentages before the fear training. (**B**) Decreased freezing percentages in HI mice than sham mice in the contextual tests at 24 h after fear training. Percentages of freezing responses were analyzed and acquired in Packwin 2.0 (Panlab, Harvard Apparatus). Data are shown as the mean and SEM for each group. Two-way repeated ANOVA followed by fisher multiple comparison test, *P < 0.05, **P < 0.01. N = 8 mice in sham and 14 in HI group.

In the fear conditioning test, mice will form a fear association between electronic foot shocks and contextual cues, and show freezing behaviors in training chambers. This process requires the hippocampal function of the contextual information processing and involves other brain areas such as the amygdala^39,40^. We first trained the mice with 5 cycles of electronic foot shocks in a chamber to form a fear association between electronic foot shocks and contextual cues. In the sham and stroke groups, mice behaved comparably in the training phase with electronic foot shocks of an intensity of 0.3 mA, displaying similar increasing percentages of the freezing time (Fig. 6A; two-way repeated ANOVA, for groups: F (1, 7) = 3.750, *P* = 0.09402; for trainings: F (5, 35) = 71.31335, *P* = 2.37 × 10^–17^; for the group x training interaction: F (5, 35) = 0.72517, *P* = 0.60916). Both groups of mice showed high percentage of freezing responses after 5 cycles of electronic shocks (by fisher post hoc, *P*_Hab_ = 0.65671, *P*_1_ = 0.42485, *P*_2_ = 0.67922, *P*_3_ = 0.4995, *P*_4_ = 0.0816, *P*_5_ = 0.14154), which suggested that the HI mice could still sense the shocks and acquire the fear behaviors after training. Next in the fear retention phase, we placed the mice in the same training chamber to test their freezing responses without foot shocks. In the contextual test as shown in Fig. 5B, two-way repeated ANOVA analysis showed that both group and time had significant effects on the freezing percentages of mice (Fig. 6B; for groups: F (1, 7) = 5.47487, *P* = 0.05186; for time: F (5, 35) = 49.3979, *P* = 4.54 × 10^–7^; for the group x training interaction: F (5, 35) = 1.03078, *P* = 0.38228). Initially both the sham mice and HI mice showed strong freezing responses in the training chamber without foot shocks, but the freezing percentage of the HI mice significantly decreased at 24 h after training (by fisher post hoc, P_P0_ = 0.05325, P_P24h_ = 0.0075, P_P72h_ = 0.1535). These results showed that the recall, but not the formation, of new fear memory association with contextual cues was impaired by cerebral ischemia and hypoxia, further supporting the weakened function of hippocampus in the adult HI model.

## Discussion

Previous studies have shown that the ischemia-followed hypoxia rapidly induces clinically similar thrombosis and early brain damage in adult HI rodent models. Here, we extended these findings and showed long-term neuronal losses and prominent reactive astrocytes and microglia present in the ipsilateral striatum and hippocampus after unilateral common carotid artery occlusion and hypoxia^8^. More importantly, in a series of behavioral tests, we demonstrated that the HI mice had an increased number of foot faults in the tapered beam test and a decreased hanging time in the wire hanging test. The HI mice needed more time and swam longer distances to find the platform in the Morris water maze test and showed fewer percentages of freezing behaviors in the contextual test after fear conditioning training. Together, these results showed motor and cognitive behavioral deficits in line with the long-term pathological changes found in these adult HI mice.

The permanent HI model in adult mice was previously reported to induce rapid microvascular thrombosis and obvious infarcted volume in mice, beginning in the middle cerebral artery (MCA) territory and spreading to the anterior cerebral artery (ACA) and part of the posterior cerebral artery (PCA) territories^8,41^. The ischemia-induced injuries in the MCA territory were mainly involved with part of the parietal–temporal cortex and the striatum^42,43^. We found prominent neuronal losses in the striatum after HI conditions, which might partially explain the motor behavioral deficits. The blood supply to the dorsal hippocampus is provided by the PCA and the anterior choroidal artery (AchA, rising from the internal carotid artery)^8,42^, and consistently, we also found prominent long-term neuronal losses in the pyramidal and granule layers of the hippocampus in adult HI mice. Furthermore, our data showed the long-term presence of reactive astrocytes and microglia in the injured area. These reactive glial cells proliferated and formed glia scar-like structures. They displayed typical reactive glial morphology with enlarged soma and processes and are thought to produce inflammatory responses and contribute to secondary long-term neuronal degeneration after stroke^15,19,20,44^.

Neuronal losses in the striatum lead to motor disorders in ischemic mice^45–47^. Consistently, we found the tapered beam and wire hanging tests could distinguish the motor deficits in the short and long term after HI condition, as demonstrated by reduced limb function in the HI mice (Fig. 4E,F). But our data show that the OFT and rotarod test could not distinguish the motor deficits inconsistent with part of previous reports^12,48^. The HI neonatal mice had a decreased mean velocity and abnormal hyperactive exploratory behaviors in the OFT and reduced holding time in the rotarod test at 7 weeks after HI conditions^12^. The inconsistency with our results might be due to the different time windows for behavioral tests or the severity of brain injury among different animal models^49–57^. On the other hand, the tapered beam and wire hanging tests could detect the early-stage and long-term motor deficits of adult HI mice, which are also used in the MCAO model^58^. In the beam test, the transient or permanent MCAO mice showed the increased foot faults at 7–14 days after surgery^59,60^. The wire hanging test detected the deficits only in the aging MCAO mice within 3 days post stroke^61^, but could not distinguish in the young MCAO mice within 9 days^61^ and 22 days^62^ post stroke. Therefore, we suggest that the tapered beam and wire hanging tests, but not the OFT and rotarod, are more suitable for examining the motor deficits of adult HI mouse models in future preclinical studies and drug screening.

Cognitive impairment and memory dysfunction are common symptoms after a stroke. Consistently, we found both structural and functional hippocampal deficits in adult HI mice. HI mice were slower learners in the water maze test. They spent more time and took longer distances to find the landing platform during training, similar to those found in the neonatal HI model^12^. But there was no significant difference between the sham and HI mice in the probe test, possibly due to the unclear preference to the target quadrant of sham mice. In previous publications, the control mice could spend most of the total time searching the platform in the target quadrant^23,48,63,64^, but in our study, sham mice only spent about 27.7% of the total time in the target area and switched to other adjacent quadrants (separately 32.9%, 20.7% and 18.6%), which was beyond our expectation. The other possible reason for the unexpected results of the probe test is the compensatory function of the contralateral side and/or overtraining of stroke mice in the tests^65–71^, leading to a duration comparable with sham mice. Furthermore, in the fear conditioning test, we found that both the sham control and HI mice could sense the electrical shocks and acquire the fear behaviors after training. However, the contextual fear memory is significant impaired in the HI mice, further supporting the weakened function of hippocampus in the adult HI model. Similar deficits in contextual fear conditioning were reported in neonatal HI rats^72^ and MCAO mice^73^. Overall, the adult HI mode shows consistent hippocampus-related behavioral deficits and could be a reliable model for assessing cognitive dysfunction associated with stroke.

As a leading cause of death and long-term disability worldwide, stroke has been a huge burden on society. Many promising drugs showed benefits on animal models, but limited success on humans. Thus, it urgently needs a more relevant animal model and better practice in preclinical studies^6,7^. Compared with other commonly-used stroke models including the thromboembolic, the photothrombotic and the filament-MCAO models, the adult HI model has several advantages. First, by the permanent RRCA ligation and hypoxia, the HI model displays the clinical spontaneous thrombosis by endothelial cells and fibrin-mediated inflammatory responses in cerebral blood vessels^8^. In the thromboembolism model, brain injury is induced by the injection of endothelin-1 or thrombin and critically depends on the potency of the peptides to form the platelet coagulation^74,75^. The photothrombotic injury is caused by the irradiation at certain wavelength after the injection of photosensitive dyes, Rose Bengal or Erythrosine B^76^, and the filament-MCAO model requires the exogenous filament to form the thrombosis in cerebral arteries. The latter three models all need the exogenous emboli or chemicals instead of the spontaneous thrombus formation^77–79^. Second, the HI model represents a clinic progress of a primary cytotoxic excitotoxicity followed by thrombosis-associated inflammation and apoptosis within a few hours^8^. But the damage in photothrombotic model evolves very rapidly and induces both processes simultaneously, leading to an intense development of necrosis in the infarction core with limited ischemic penumbra^80,81^. Specifically, the photochemical could produce certain blood-borne factors and acute physiological changes by significantly affecting the blood–brain barrier function, which may have direct impact on the behavioral performances^82^. In filament-MCAO model, the mechanical occlusion barely reflect the hemodynamics after thrombolytic reperfusion and are not suitable for thrombolysis studies^83^. Third, in the HI model, neuronal losses exist in both superficial and deep brain areas including the striatum and hippocampus, whereas the infarct of photothrombotic model is mainly in the superficial brain area^76^. In the filament-MCAO model, the infarct mainly exists in the cortex and striatum, and is less affected the hippocampus in part of the publications^84^. Lastly, the surgery is simple and easy to perform in the HI model, which greatly facilitates the application among labs. The filament-MCAO model usually needs proficient surgical skills and has a risk of vessel rupture and hemorrhage in the surgery. The other two models need the craniotomy to receive the chemical injection or irradiation, and have a risk of the intracranial infections.

In conclusion, this study demonstrates long-term neuronal losses and chronic gliosis in adult HI mice which are associated with both motor and cognitive deficits. We believe that the adult HI mice model could be useful for preclinical stroke research and drug screening.

## Materials and methods

### Animal care

Male C57BL/6J mice were purchased from Guangdong Medical Laboratory Animal Center and carefully raised at the animal house of South China Normal University. Food and water were provided ad libitum. The care and use of laboratory animals followed the ethical guidelines of the Ethics Committee for Animal Research at South China Normal University and were in accordance with the guide of the National Institutes of Health. All experimental protocols were approved by the Ethics Committee for animal research at South China Normal University.

### Stroke model

Following a previous procedure^8^, 12–14 week old C57BL6 mice were induced anesthesia with 2% isoflurane and maintained by 1.5% isoflurane. In stroke modelling, we first ligated the right common carotid arteries with two knots using 6-0 silk. Rectal temperature was controlled at 37.5 ± 0.5 °C by a temperature controller with heating pads during surgery. After 2 h of recovery, the mice were subjected to hypoxia (7.5% oxygen balanced with 92.5% nitrogen) for 40 min in a chamber (25 × 12 × 18 cm). During the hypoxia, the oxygen concentration was maintained at 7.5 ± 0.5% by an oxygen detector and the room temperature was maintained at 24 ± 1 °C. The stroke mice were scored about 30 min after hypoxia as previously reported^8^ with slight modifications. These scores are: 1. no detectable deficits; 2. ptosis of eyelid ipsilateral to the ligated CCA; 3. mice persistently walk in circles; 4. mice lie nearly motionless on the contralateral side/paralysis; 5. animal dies. In our trials, 15 mice showed a circling performance, 2 lay motionless and 2 had ptosis of the eyelid after stroke. For sham control mice, we exposed the right common carotid arteries without ligation or hypoxia. After surgeries, the animals were returned to their home cages. For post-surgery care, special food of soft jelly was additionally provided to replenish fluids and encourage eating during the first week. Daily body weights of mice were carefully monitored in our experiments. On average, the stroke mice body weights decreased about 10% at 3D after stroke (PStr3D), but recovered to similar weights prior to surgery at PStr10D.

### Immunohistochemistry

The mice were anesthetized with 10% urethane, then transcardially perfused with 0.01 M phosphate-buffered saline (PBS; PH 7.2–7.4) to wash off the blood, followed by 4% paraformaldehyde (PFA) to fix the tissue. The brains were extracted and post-fixed in 4% PFA at 4 °C overnight. After being embedded sequentially in 15% and 30% sucrose solutions, the brains were sectioned into 40 μm slices by a freezing microtome (Leica, Germany). For immunohistochemistry, the sections were washed 3 times (5 min each) in 0.01 M PBS and blocked with 5% donkey serum in 1% Triton X-100 (in PBS; 1%PBST) for 1 h at room temperature (RT). Primary antibodies (NeuN, rabbit, diluted at 1:1000, Merck Millipore, USA; GFAP, rat, 1:1000, Thermo Fisher Scientific, USA; MAP2, chicken, 1:1000, Abcam, USA; IBA1, rabbit, 1:1000, Wako Chemicals, USA) were incubated with brain slices overnight at 4℃ diluted in 0.3%PBST containing 5% normal donkey serum. After washed in PBS, the slices were stained with appropriate secondary antibodies (Diluted at 1:2000, Thermo Fisher Scientific, USA) for 1.5 h and later with DAPI (Diluted at 1:2000, Sigma Aldrich, USA) for 15 min at RT. After washed in PBS, the slices were covered with an anti-fading mounting solution (Thermo Fisher Scientific, USA), and photographed with the fluorescent microscope (Nikon Eclipse Ni, Japan) and the EVOS microscope (Thermo Fisher Scientific, USA).

Our quantitative pathological data are based on 6 pairs of mice (sham vs HI), in which 3 were from the pilot experiments specifically designed for pathological confirmation and 3 from behavioral groups on which similar series of sequential sectioning were made. For the long-term lesion extension, we used 5 stroke mice (2 in the pilot and 3 in behavioral groups) and chose 4 representative brain slices spanning from + 0.4 to − 2.5 mm to the bregma from one set of brain slices in each mouse. The slices from the sham and stroke groups were in comparable and fixed coordinates to show the brain damage. The injured area is defined with the area with obvious GFAP^+^ gliosis, which persists in damage area after stroke^25–27^. The positive GFAP^+^ area was quantified by a pixel threshold on 8-bit converted images using Image J 2.0.0 (FIJI, National Institutes of Health, USA) and showed the percentage of injured area ratio with total area^26^. To examine the status of glial cells, we also chose 3 brain slices in similar coordinates from 3 pairs of mice in behavioral tests. The data were collected within three fields in striatum and one field in each sub-region of hippocampus per slice. The GFAP^+^ and IBA1^+^ fluorescence intensities are the mean fluorescence intensities, which equal the mean values of the object minus the mean values of the background^85^. The morphological analysis of soma area is quantified in Image J 2.0.0 (FIJI) according to previous protocols^86,87^ and collected with 15 cells in each slice.

### Fluoro-Jade B (FJB) staining

FJB staining was used to detect neuron degeneration in adult HI mice. After drying on a slide warmer for 30 min at 45 °C, the slices were rinsed in 80% ethanol containing 1% sodium hydroxide for 2 min, 70% ethanol for 2 min and washed twice in water. The tissue was incubated in 0.06% KMNO_4_ for 10 min at RT and washed 3 times in water. Then the slices were incubated in 0.008% Fluoro-Jade B solution (Merck Millipore, USA) containing 0.1% acetic acid for 20 min at RT and kept from lights in the following procedures. After being washed 3 times in water, the slices were dried on the slide warmer again at 45 °C and rinsed in xylene for 5 min at RT. The slides were covered with DPX mounting solution (Sigma Aldrich, USA) and observed with the fluorescent microscope under 488 nm exciting lights.

### Behavioral tests

*The open field test* detects the general spontaneous activity of the mice^29,30^. In this test, the mice could move freely in an open-field apparatus consisting of a square plastic chamber (50 × 50 × 50 cm) and were recorded by a computer system for 10 min (Zhenghua, China). The total distance, mean velocity, and time spent in the central zone of the locomotion were further analyzed. The test was performed only at 21-day after stroke.

*The rotarod test* measures motor coordination and balance in mice^30,31^. In this test, the mice had to consistently walk on a rotating rod to keep from falling (Yiyan, China). The latency to fall (holding time) was recorded to evaluate motor functions. Mice were pre-trained for 3 days with 2 trials per day (15 min per trial), and tested on 1 day pre stroke, 2 days and 21 days’ post stroke. As in previous reports^12^, the rotating speed was 20 rotations per minute (rpm) on the first training day and 30 rpm for the following days. Mice which could not hold on the rod for over 600 s after training were excluded from the test^23^. Means of the trials were further analyzed by one blinded partner.

*The tapered beam test* examines the dysfunction of the mice’s hind limbs^31^. The mice were encouraged to walk across a width-tapered beam from the widest end to a ‘safe’ dark box at the narrowest end. The beam was 1 m in length and 2.5–0.5 cm tapered in width, with underhanging ledges 0.5 cm in width placed on both sides of the beam (SansBio, China)^88,89^. The touching on the ledge of the contralateral hind limb was counted as a fault. The mice were trained for 3 days before stroke and tested at 1 day pre stroke, and 1 day and 21 days’ post stroke. Each mouse was given 3 trails per day and stayed in the dark box for 20 s in each trial. Behavioral performances were videotaped by one video system. A blinded observer watched the videos and analyzed the means of faults in 3 trials. The individuals would be excluded in the test whose fault numbers were > 6.75 on average^23,90,91^.

*The wire hanging test* examines the forelimb motor strength of mice^30^. In this test, the mice were trained to suspend their bodies from a steel wire (2 mm in diameter) with only their forelimbs (SansBio, China). The wire was held with two posts 40 cm above a soft pillow^29,62^. The mice were trained for 2 days with 3 trials per day and tested on same days as the rotarod test. The time until the mouse fell (holding time) was recorded and the averages of three trials were further analyzed by one blinded partner. Mice which could not hold for over 18 s after training were excluded from the test^23,92^.

*The Morris water-maze test* examines the spatial learning function of mice. The mice were trained to find a hidden platform to escape from the water via visual clues on the tank wall (Zhenghua, China)^29,32,33^. The diameters of the tank and the hidden platform were 120 cm and 15 cm, respectively. The water was obscured and stained white by non-toxic dye. The temperature was maintained by 19.0 ± 2.0 °C and the water surface in the tank was 1 cm higher than the height of the platform. We performed this test according to the previous protocol^34^. Before training, the mice were gently placed in water for the adaptation of water environment and we screened out the individuals that could not balance in water. In the training, a mouse was released in one random start position (4 positions in all) facing the tank wall, and the performance was recorded by computers. The longest time was 60 s in each trial. The mouse would be guided and stayed on the platform for 15 s if it could not find the target within 60 s. The training lasted for 5 days with 4 trials per day. On the probe day, the platform was removed, and the mice could swim in the pool for 60 s. The parameters in the training and probe test were further analyzed by one blinded partner.

*The fear conditioning test* examined the ability of mice to learn and remember the association between environmental cues (the training context in a soundproof chamber) and an aversive experience (mild electronic foot shocks)^30^. The conditioning apparatus was controlled with software (Packwin 2.0, Panlab, Harvard Apparatus, USA). Each mouse was individually placed in a dimly illuminated chamber and allowed to move freely for 3 min (habitation/hab). After habituation, mice received foot shocks of 0.3 mA for 2 s followed by one 18 s interval, and then a second foot shock with 5 cycles in all. After the last shock, mice were kept in chamber for another 3 min, and then returned to their home cages. In the contextual test, the mice were placed in the same training chamber for 5 min without any shocks (at 24-h and 72-h after fear training). The freezing duration was recorded in the training and contextual tests. The percentage of freezing responses (freezing duration/total × 100%) was further analyzed by one blinded partner.

### Statistical analysis

Data were shown as the mean ± SEM and plotted in Prism 8.0 (GraphPad, USA). Two-way repeated ANOVA followed by fisher post hoc analysis in Origin 2018 (OriginLab, USA) and unpaired t-test in Prism 8.0 (GraphPad, USA) was used to analyze the data. *P* value < 0.05 was considered statistically significant.

## Supplementary information

Supplementary Figure 1.
