# Supplementary material for: Deficits in motor and cognitive functions in an adult mouse model of hypoxia-ischemia induced stroke
Source: Sci Rep. 2020 Nov 26;10:20646. doi: 10.1038/s41598-020-77678-8 (PMC7692481; doi:10.1038/s41598-020-77678-8)
Supplement: Supplementary file 1 — Supplementary Figure 1. [file 41598_2020_77678_MOESM1_ESM.docx]

**Deficits in Motor and Cognitive Functions in an Adult Mouse Model of Hypoxia-Ischemia Induced Stroke**

Li Feng^1#,^ *, Chun-Xia Han^1#^, Shu-Yu Cao^2^, He-Ming Zhang^2,^ * and Gang-Yi Wu^1^

*^1^ School of Life Sciences, South China Normal University, Guangzhou, 510631, China.*

*^2^ Institute for Brain Research and Rehabilitation, South China Normal University, Guangzhou, 510631, China.*

*Corresponding authors: [fenglifl0612@126.com](mailto:fenglifl0612@126.com) and [d_zhm@163.com](mailto:d_zhm@163.com)

^#^ Co-contribution authors

**Supplementary Figure 1 Injured area in the whole brain of adult HI mice.**

(A) Neuron losses (stained with a neuronal marker, NeuN, green) in motor and sensory cortex 4 weeks after hypoxia and ischemia. The brain areas were defined with the help of the Mouse Brain in Stereotaxic Coordinates^1^. (B) Representative images of the reactive microglia (stained by the microglial marker, IBA1, red) in the whole brain. The reactive microglia have contributions to the secondary brain injury in the long term after HI conditions. The images were photographed by an inverted fluorescent microscope (EVOS FL Auto). Scale bar in (A) is 500μm and (B) 1mm.


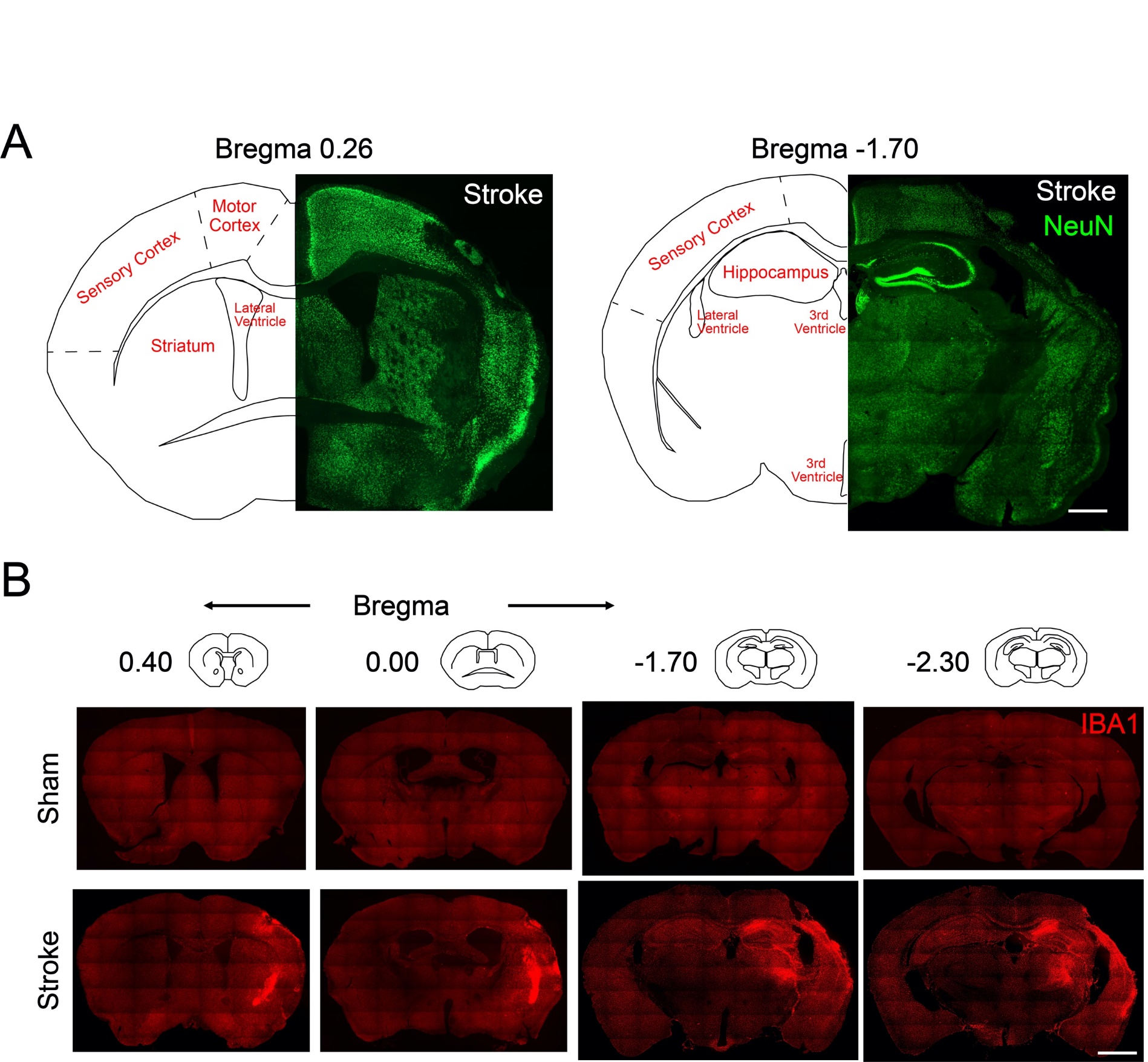


**Reference**

1. Franklin, K. B. J. & Paxinos, G. *The Mouse Brain in Stereotaxic Coordinates*. (Academic Press, 1997).
